# Efficacy of Long-Term Use of Azithromycin in the Management of Cystic Fibrosis in Pediatric Patients with or Without *Pseudomonas aeruginosa*: A Systematic Review and Meta-Analysis Article

**DOI:** 10.3390/medicina61040653

**Published:** 2025-04-02

**Authors:** Hassan Al-shehri, Dana Albassam

**Affiliations:** Department of Pediatrics, College of Medicine, Imam Mohammad Ibn Saud Islamic University (IMSIU), Riyadh 13317, Saudi Arabia; daalbassam@imamu.edu.sa

**Keywords:** azithromycin, cystic fibrosis, children, pulmonary function, *Pseudomonas aeruginosa*

## Abstract

*Background and Objectives:* In the present systematic review and meta-analysis, we aimed to discover the overall efficacy of azithromycin in children with cystic fibrosis (CF) and with or without *Pseudomonas aeruginosa* infection, specifically regarding its effect on respiratory parameters such as forced expiratory volume in 1 s (FEV1) and forced vital capacity (FVC) in addition to its effect on exacerbations and the need to use additional antibiotics. *Materials and Method:* We conducted this systematic review and meta-analysis by searching for all eligible articles on PubMed, Web of Science, and Scopus published between inception and September 2024. We used the following search strategy for our searching process: “Cystic fibrosis” AND “Azithromycin” and “Children” OR “Pediatric” OR “Infant”. We conducted the meta-analysis by pooling the mean difference (MD) and comparing the continuous variables and odds ratio (OR) for dichotomous variables at 95% confidence intervals (CI), at a *p*-value of 0.05. *Results:* Azithromycin was observed to be associated with increased FEV1 compared with the control, showing an MD of 1.91 (95% CI: 1.09, 2.74, *p* < 0.00001) and non-significant heterogeneity. However, no significant difference was observed between azithromycin and control groups regarding FVC with MD = 0.62 (95% CI: −0.01, 1.25, *p* = 0.06). Compared with the control group, azithromycin was significantly associated with lower risk and a lower number of exacerbations, with OR = 0.48 (95% CI: 0.34, 0.67, *p* < 0.0001) and MD = −0.82 (95% CI: −1.32, −0.33, *p* = 0.001), respectively, with non-significant heterogeneity. Regarding the need for new antibiotic usage, azithromycin showed a significantly lower need, with OR = 0.35 (95% CI: 0.13, 0.94, *p* = 0.04), I^2^ = 75%, *p* = 0.02. No significant difference was observed between both groups regarding hospitalization rate, with OR = 0.88 (95% CI: 0.55, 1.4, *p* = 0.59). *Conclusions:* This systematic review and meta-analysis showed the efficacy of azithromycin in pediatric patients with CF, as it improved lung function by increasing FEV1, reduced exacerbations of CF, which is the most common symptom of CF that leads to mortality, and reduced the number of antibiotics that needed to be administered to patients with CF, which reduces the risk of antibiotic resistance. Therefore, the long-term use of azithromycin is recommended for pediatric patients with CF as part of their treatment regimen.

## 1. Introduction

Cystic fibrosis (CF) is an autosomal recessive multisystem disorder characterized by severe symptoms in the respiratory, urogenital, and gastrointestinal systems. The cumulative symptoms significantly impair the quality of life for many individuals and necessitate several hospitalizations during the course of their illness. Research indicates that most hospitalizations result from pulmonary symptoms, thereby exacerbating the disease’s burden [1,2]. It is the most prevalent cause of early mortality among autosomal recessive illnesses in the global Caucasian population [3].

CF is linked to mutations in the CF transmembrane conductance regulator (CFTR) gene situated on the long arm of chromosome 7. CFTR encodes a protein present in the epithelial cells of the lungs, sweat glands, pancreas, intestines, and liver. It is part of the Adenosine triphosphate (ATP-) binding cassette transporter family and operates as a Cyclic adenosine monophosphate (cAMP) dependent chloride channel that enables Cl^−^/HCO_3_^−^ exchange in exocrine epithelia [4]. It functions to sustain an alkaline pH and dilute fluid secretions [5]. Mutations in CFTR result in dehydrated secretions and hyperviscous mucus, leading to a multisystem disorder that significantly impacts the respiratory, gastrointestinal, and hepatobiliary systems. The fundamental flow in ion transport creates a continuous loop of compromised airway clearance, persistent endobronchial infection, and excessive inflammatory response in the lungs [6]. Chronic high-intensity inflammation causes irreversible structural damage to CF airways and deteriorates lung function, ultimately leading to respiratory failure and mortality. Numerous impaired inflammatory responses have been associated with CFTR loss, encompassing innate and adaptive immunological dysregulation, anomalies in cell membrane lipids, different transcription factor signaling deficiencies, and altered kinase and toll-like receptor responses [7]. Lung inflammation in CF is characterized by neutrophils that secrete oxidants and proteases, including elastase. Neutrophil elastase in airway secretions precedes bronchiectasis and correlates with the decline in lung function and respiratory exacerbations in CF [8].

Pulmonary exacerbation is the most prevalent acute occurrence in patients with CF [9]. Clinical trials have shown *Pseudomonas aeruginosa* as the most often implicated bacteria in acute exacerbations [10,11].

Macrolides, including azithromycin, have been extensively researched in patients with CF, both with [12] and without [13] *Pseudomonas aeruginosa* infection. This research consistently demonstrates a clinically significant decrease in the incidence of pulmonary exacerbation for up to 6 months. Macrolides have not exhibited bactericidal activity against *Pseudomonas aeruginosa* and are not deemed suitable as a singular or first-line antibiotic for eradication therapy. Macrolides exhibit anti-inflammatory properties [14] and may diminish *Pseudomonas aeruginosa* biofilm formation [15], which could partially elucidate their influence on pulmonary exacerbations. In the present systematic review and meta-analysis, we aimed to discover the overall efficacy of azithromycin in children with CF with or without *Pseudomonas aeruginosa* infection, specifically regarding its effect on respiratory parameters such as forced expiratory volume in 1 s (FEV1) and forced vital capacity (FVC), in addition to its effect on exacerbations and the need to use additional antibiotics.

## 2. Materials and Methods

### 2.1. Database Searching and Screening

By adhering to the Preferred Reporting Items for Systematic Reviews and Meta-Analyses (PRISMA, File S1) guidelines [16], we conducted this systematic review and meta-analysis by searching for all eligible articles on PubMed, Web of Science, and Scopus published between inception and September 2024. We used the following search strategy for our searching process: “Cystic fibrosis” AND “Azithromycin” and “Children” OR “Pediatric” OR “Infant”.

### 2.2. Eligibility Criteria

We selected studies based on the following inclusion criteria: studies published without time frame limitations, randomized controlled trial (RCT) studies, and observational cohort studies comparing the use of azithromycin against a control group (those receiving no azithromycin) or a placebo in pediatric patients with CF. We excluded reviews, case reports, studies with no comparison groups, and studies analyzing drugs other than azithromycin.

### 2.3. Screening

For the screening process, the search results were uploaded on Rayyan [17] to facilitate title and abstract screening. The eligible articles underwent full-text screening after the previous step to examine the eligibility of the whole article. Any disagreement was resolved by consensus, and, if it persisted, it was referred to the senior author.

### 2.4. Data Extraction

Using Microsoft Excel spreadsheets, we conducted the process of data extraction to extract the baseline data and outcomes of interest. Regarding the baseline data, we collected data on study design, population, age, gender, sample size, follow-up, and the use of other drugs. For the outcomes, we extracted data on changes in FEV1 and FVC, exacerbations, hospitalization, and the use of antibiotics.

### 2.5. Quality and Risk of Bias Assessments

Utilizing the Newcastle–Ottawa Scale (NOS), which rates each study from 0 to 9 stars, quality evaluation for the observational cohort studies was carried out. With the exception of the comparability question, the answer to which can receive zero, one, or two stars, each answer can be given a star. A study is rated as low quality if it receives 0–3 stars, moderate quality if it receives 4–6 stars, and high quality if it receives 7–9 stars [18]. We also utilized the Cochrane risk-of-bias instrument (Rob-2) [19], comprising five domains with associated sets of questions. These domains include randomization, deviations from intended interventions, outcome measurement, missing outcome data, and outcome selection of the reported result. A study can only be considered to have an overall low risk of bias if each of the five domains is classified as low risk individually. If concerns are expressed in any domain, then the study is considered to have problems. Additionally, if any domain is judged to be at high risk or if multiple domains raise red flags, the study is labeled as having a high risk of bias.

### 2.6. Statistical Analysis

Using Review Manager Version 5.4 [20], we conducted a meta-analysis by pooling the mean difference (MD) and comparing the continuous variables and odds ratio (OR) for dichotomous variables. The random effect model was used for heterogenous variables and the fixed effect model was used for homogenous ones, with 95% confidence intervals (CI) and a *p*-value of 0.05. The heterogeneity was assessed using the I^2^ and a *p*-value of 0.05.

## 3. Results

### 3.1. Search Results and Screening

**Figure 1:**
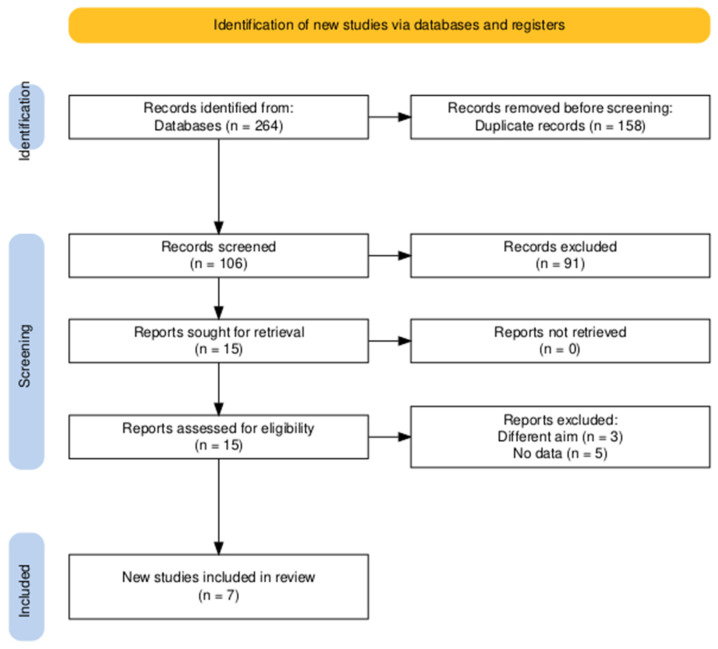
PRISMA flow diagram of the searching and screening processes.

The applied search strategy resulted in a total of 264 articles with 158 duplicates. After title and abstract screening, 15 articles were found to be eligible for full-text screening, and a final 7 articles were eligible to undergo systematic review and meta-analysis [13,21,22,23,24,25,26] (Figure 1).

### 3.2. Quality and Risk of Bias Assessments

**Figure 2:**
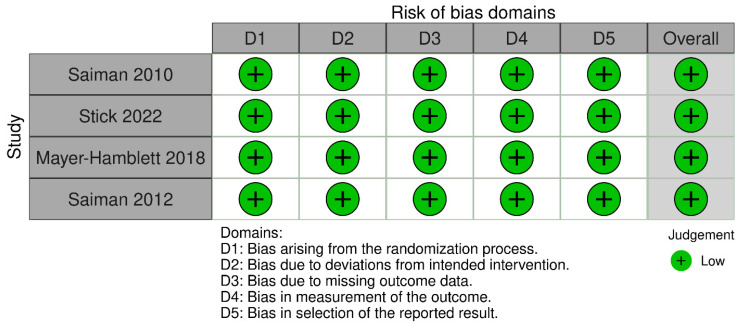
Risk of bias assessment of randomized controlled trials using Rob-2 [13,21,24,26].

According to Rob-2, the four included RCTs were found to have a low risk of bias across all domains (Figure 2). The three included cohort studies were deemed to have high quality according to the NOS (Table 1).

### 3.3. Baseline Characteristics

We included seven studies in the present systematic review and meta-analysis, comparing the use of azithromycin against a control group (no azithromycin) or a placebo in pediatric patients with CF, who either experienced or did not experience *Pseudomonas aeruginosa* infection. Four studies were RCTs, while the other three were cohort studies. Follow-up ranged from 5.6 to 36 months. The mean age of the patients ranged from 3.6 months to 18 years old. Baseline characteristics are shown in Table 2.

### 3.4. Statistical Analysis

**Figure 3:**
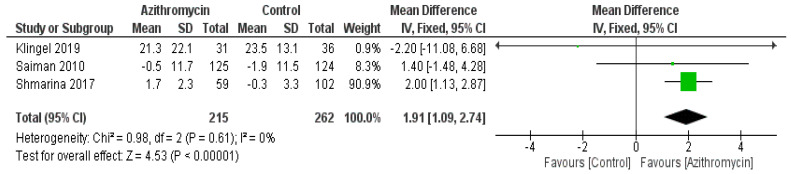
A comparison between the azithromycin and control groups regarding FEV1 change from baseline. The diamond shape is the forest plot, and the squares represent the effect size of each study [13,23,25].

**Figure 4:**
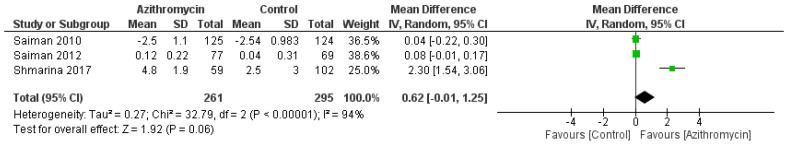
A comparison between the azithromycin and control groups regarding FVC change from baseline. The diamond shape is the forest plot, and the squares represent the effect size of each study [13,23,26].

Azithromycin was observed to be associated with increased FEV1 compared with the control, showing an MD of 1.91 (95% CI: 1.09, 2.74, *p* < 0.00001) and non-significant heterogeneity. However, no significant difference was observed between the azithromycin and control groups regarding FVC, with an MD = 0.62 (95% CI: −0.01, 1.25, *p* = 0.06) (Figure 3 and Figure 4).

**Figure 5:**
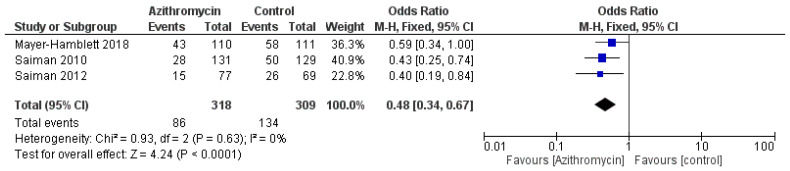
A comparison between the azithromycin and control groups regarding the risk of exacerbations. The diamond shape is the forest plot, and the squares represent the effect size of each study [13,21,26].

**Figure 6:**
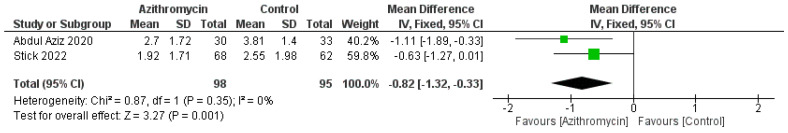
A comparison between the azithromycin and control groups regarding number of exacerbations. The diamond shape is the forest plot, and the squares represent the effect size of each study [22,24].

Compared with the control group, azithromycin was significantly associated with a lower risk and a lower number of exacerbations, with an OR = 0.48 (95% CI: 0.34, 0.67, *p* < 0.0001) and an MD = −0.82 (95% CI: −1.32, −0.33, *p* = 0.001), respectively, with non-significant heterogeneity (Figure 5 and Figure 6).

**Figure 7:**
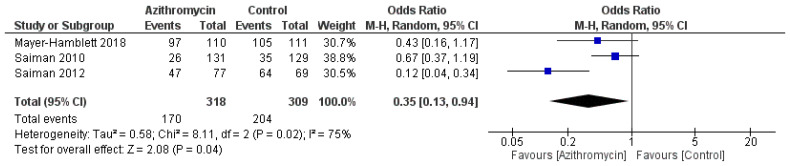
A comparison between the azithromycin and control groups regarding additional antibiotic usage. The diamond shape is the forest plot, and the squares represent the effect size of each study [13,21,26].

**Figure 8:**
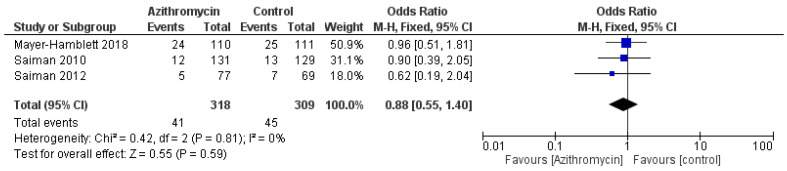
A comparison between the azithromycin and control groups regarding hospitalization rates. The diamond shape is the forest plot, and the squares represent the effect size of each study [13,21,26].

Regarding the need to use additional antibiotics, azithromycin resulted in a significantly lower need, with an OR = 0.35 (95% CI: 0.13, 0.94, *p* = 0.04), I^2^ = 75%, *p* = 0.02 (Figure 7). No significant difference was observed between both groups regarding hospitalization rates, with an OR = 0.88 (95% CI: 0.55, 1.4, *p* = 0.59) (Figure 8).

## 4. Discussion

The current systematic review and meta-analysis aimed to evaluate the efficacy of azithromycin use in pediatric patients with CF. The present analysis showed the efficacy of azithromycin in increasing the FEV1, which indicates improved pulmonary function, in decreasing exacerbations, which is the main complaint of patients with CF, and in decreasing the need to use additional antibiotics, which is an important parameter to avoid associated adverse events, mainly antibiotic resistance.

The mechanism of action of azithromycin in CF remains unclear. Previous research [12,27,28] has indicated that azithromycin does not eliminate CF germs. Previous in vitro investigations indicated that azithromycin reduced the expression of proinflammatory cytokines, while exhibiting inconsistent effects on anti-inflammatory cytokines and modifying bacterial traits, including the pili, flagella, exoproducts, and the quorum-sensing ability of *Pseudomonas aeruginosa* [29,30]. Azithromycin has demonstrated efficacy in diminishing neutrophil recruitment in non-CF animal models [31] infected with *Pseudomonas aeruginosa* and in reducing neutrophilia and interleukin (IL)-8 levels in people with bronchiolitis obliterans syndrome [32]. In CF airway epithelial cells, azithromycin decreased IL-8 transcription and protein production while decreasing the DNA binding of the IL-8 transcriptional regulators nuclear factor-kappa B (NF-κB) and activator protein-1 (AP-1) [33]. In F508 homozygous CF mice, azithromycin has demonstrated efficacy in diminishing baseline inflammation and inflammation produced by *Pseudomonas aeruginosa* lipopolysaccharide [34]. Nonetheless, empirical evidence regarding the anti-inflammatory properties of azithromycin in CF is limited.

The impact of prolonged low-dose azithromycin treatment on the airways can be elucidated by many theories. The predominant mode of action examined is the antibacterial efficacy of the medication in both intracellular and extracellular environments. Azithromycin, a 15-membered ring azalide chemical, is produced from erythromycin. Its distinctive structure facilitates improved intracellular penetration and tissue accumulation [35]. Azithromycin is recognized for its anti-inflammatory and tissue reparative properties. Reported additional effects of macrolides include the inhibition of proinflammatory mediator release, reduced neutrophil infiltration in the lungs, the modulation of mucus secretion, and the modification of the biofilm matrix [36]. This demonstrates that macrolides significantly advantage the azithromycin group by suppressing the proliferation of *Pseudomonas aeruginosa*, altering host defenses, and interacting with several cell types in the airways to diminish inflammation [35]. Saiman and colleagues [12] observed that sputum neutrophil elastase activity escalated in the placebo group after six months, but it remained unchanged in the azithromycin group. In contrast, Equi and colleagues [27] were unable to record any alterations in airway IL-8 and neutrophil elastase levels in the subgroup of patients who underwent sputum testing. This gap can be attributed to the challenges of acquiring valid measures of inflammatory mediators in the sputum of patients with CF. Future studies should implement standardized sputum collection and processing protocols to ensure consistency, use biomarker normalization techniques (e.g., expressing inflammatory mediator levels relative to total protein content or sputum volume) to account for dilution effects, and adopt longitudinal sampling to capture fluctuations in inflammation over time. Additionally, employing advanced analytical techniques, such as high-sensitivity assays or mass spectrometry, can enhance biomarker detection and quantification. Exploring alternative biofluids, like exhaled breath condensate or bronchoalveolar lavage fluid, may also provide complementary insights into airway inflammation. These refinements can help improve the reliability of inflammatory mediator measurements and clarify the effects of azithromycin on CF airway inflammation. Culic et al. [37] discovered that the immunomodulatory effect of azithromycin is contingent upon the duration and stages of inflammation experienced. Research conducted on both human and animal models indicates that azithromycin exerts a beneficial immunomodulatory effect when administered early during bacterial infection; however, its efficacy diminishes significantly when used later in the inflammatory process, resulting in an elevation of inflammatory mediators. This elucidates why pre-treatment is a superior technique for preventing exacerbations [22].

In addition to its well-documented anti-inflammatory and antimicrobial effects in cystic fibrosis, azithromycin has been investigated for its impact on gastrointestinal motility. Patients with CF frequently experience gastrointestinal dysfunction, including delayed gastric emptying and small bowel dysmotility, which can contribute to malabsorption and nutritional challenges [38]. Emerging evidence suggests that azithromycin may modulate gastrointestinal motility, as demonstrated by Shakir and Altaf [39], who reported that azithromycin induces migrating motor complexes in pediatric patients undergoing antroduodenal motility studies. This prokinetic effect could have clinical relevance for patients with CF and gastroparesis or small bowel dysmotility. Altered motility patterns may influence the timing and efficiency of nutrient absorption, potentially impacting the overall nutritional status of patients with CF who are already at risk of malnutrition. Furthermore, changes in gastrointestinal transit time could interfere with the effectiveness of pancreatic enzyme replacement therapy (PERT), which requires appropriate timing relative to food intake for optimal digestion and nutrient absorption. If azithromycin significantly alters small bowel transit, adjustments of PERT dosing or timing may be necessary to maintain adequate digestion and absorption.

Azithromycin use has been reported to have an impact on the gut microbiome. A meta-analysis by McDonnell et al. [40] highlighted the significant impact of macrolide antibiotics on the gut microbiome in children, particularly in reducing alpha-diversity and microbial richness. Significant reductions were seen in Bifidobacteria and Lactobacillus, and significant increases in Proteobacteria such as E. coli were also observed. This reduction can have substantial downstream effects on immune function and metabolic processes. The findings suggest that macrolide-induced gut dysbiosis may contribute to long-term alterations in host immunity, potentially increasing susceptibility to inflammatory conditions, allergies, and metabolic disorders. Therefore, this should be adequately monitored. Azithromycin has a prolonged half-life, allowing it to persist in tissues for an extended period. This pharmacokinetic property contributes to its long-term side effects including its effects on the gut microbiome, even after the discontinuation of treatment. Further research is necessary to determine the optimal strategies for mitigating these effects, such as probiotic co-administration or microbiome monitoring during prolonged azithromycin use [41].

Saiman et al. [13] reported that azithromycin was well tolerated in terms of safety. No rise in serious or nonserious adverse events was seen among subjects administered azithromycin. Nausea, diarrhea, and wheezing, previously noted to occur more frequently in the azithromycin group [12], manifested with comparable frequency in both the azithromycin and placebo groups in the current trial, affecting fewer than 10% of patients in each cohort. Conversely, cough and productive cough were less prevalent in the azithromycin group, aligning with the decrease in pulmonary exacerbations, as delineated in the current meta-analysis.

Prior research in adults has indicated a heightened risk of hearing loss [42,43] and a possible extension of the corrected QT interval (QTc) interval in patients with chronic cardiovascular illnesses [44] associated with azithromycin. While analogous findings have not been observed in the CF population [12,13,27], the extended duration of OPTIMIZE [21] facilitated a more thorough identification of potential cardio- or ototoxicity in this young, susceptible demographic via meticulous audiologic and Electrocardiogram (ECG) surveillance. The randomized, controlled segment of OPTIMIZE demonstrated no instances of temporary or permanent hearing loss or clinically significant QTc prolongation, so further affirming the safety of macrolides in this demographic. The recent literature indicates a detrimental interaction between tobramycin inhalation solution and azithromycin, both in vitro [45] and in vivo [46], as evidenced by retrospective investigations involving persons with CF chronically infected with *Pseudomonas aeruginosa*. The OPTIMIZE [21] experiment assessed a possible interaction when both medications were administered concurrently. Tobramycin inhalation solution was prescribed solely in reaction to a *Pseudomonas aeruginosa*-positive culture during the subsequent 15-month follow-up period following the initial medication. Consequently, a potential interaction may be most effectively assessed at the beginning phase of treatment for the eradication of *Pseudomonas aeruginosa*. At the conclusion of the first quarter of therapy, no significant disparity was observed in eradication rates or clinical outcomes, including lung function, between patients administered tobramycin inhalation solution with azithromycin and those receiving tobramycin inhalation solution with placebo. Consequently, in this context, they were unable to identify any adverse interaction; nonetheless, this study may not have permitted adequate time to explore a possible antagonism. Consequently, further exploration remains necessary [21].

Despite the efficacy provided by azithromycin in the long-term management of CF in pediatrics, the resistance to macrolides remains a problem. A prior meta-analysis [47] showed that the long-term use of azithromycin in chronic lung diseases was significantly associated with increased risk of resistance. Therefore, strategies to manage this risk are still needed due to the lack of data concerning this finding.

In the evolving landscape of CF management, the introduction of disease-modifying drugs (DMDs) has fundamentally altered treatment paradigms. While azithromycin has demonstrated efficacy in reducing exacerbations and improving lung function, its long-term use must be re-evaluated in light of emerging therapies that directly target the underlying CFTR dysfunction. The potential impact of DMDs on airway inflammation, infection burden, and overall disease trajectory raises important questions about the necessity and optimal duration of adjunctive macrolide therapy. Notably, the current body of literature, as included in this meta-analysis, lacks substantial data on the interplay between long-term azithromycin use and DMD therapy. Future research should focus on defining patient subgroups who may continue to benefit from azithromycin in the DMD era, ensuring a more tailored and evidence-based approach to CF management [48].

Although the current systematic review and meta-analysis have several methodological advantages, such as investigating different outcomes regarding the pulmonary function and signs and symptoms of CF, in addition to the inclusion of all available RCTs and cohort studies conducted on children with CF receiving azithromycin, some limitations still exist which warrant further investigation. These include the small number of studies for most of the outcomes and, therefore, the small sample size for these outcomes; the inadequate investigation of adverse events in the published articles; the lack of comparisons made between patients with CF and *Pseudomonas aeruginosa* and those without, which may affect the efficacy of azithromycin; and the absence of investigation of confounding factors that affect treatment results such as the use of other drugs, associated comorbidities, and the duration of treatment. Therefore, we recommend that future large-scale RCTs be conducted to investigate the risk of adverse events, the effect of *Pseudomonas aeruginosa* infection, and the role of confounding factors on the treatment outcomes. We also recommend further trials to investigate the risk of macrolide resistance and how to overcome it.

## 5. Conclusions

This systematic review and meta-analysis showed the efficacy of azithromycin in pediatric patients with CF; it improved lung function by increasing FEV1, reduced exacerbations of CF, which is the most common symptom of CF that leads to mortality, and reduced the number of additional antibiotics that needed to be administered to patients with CF, which reduces the risk of antibiotic resistance. Therefore, the long-term use of azithromycin is recommended for pediatric patients with CF as part of their treatment regimen; however, future RCTs are still warranted to determine the duration of treatment and the effect of other factors in the process of management.

## Figures and Tables

**Table 1:** Quality assessment of cohort studies using the NOS.

Study Name	Representativeness of the Exposed Cohort (★)	Selection of the Non-Exposed Cohort (★)	Ascertainment of Exposure (★)	Demonstration That Outcome of Interest Was Not Present at Start of Study (★)	Comparability of Cohorts Based on the Design or Analysis (Max.: ★★)	Was Follow-Up Long Enough for Outcomes to Occur? (★)	Assessment of Outcome (★)	Adequacy of Follow-Up of Cohorts (★)	Quality Level
Shmarina 2017 [23]	✩	✩	✩	✩	✩	✩	✩	✩	High
Klingel 2019 [25]	✩	✩	✩	✩	✩	✩	✩	-	High
Abdul Aziz 2020 [22]	✩	-	✩	✩	✩	✩	✩	✩	High

**Table 2:** Baseline characteristics of the included patients.

Study ID	Design	Population	Follow-Up (Months)	Sample Size		Age, Mean (SD) Years		Male, n (%)		Other Drugs
				AZN	No AZN	AZN	No AZN	AZN	No AZN	
Saiman 2010 [13]	RCT	Patients with cystic fibrosis uninfected with *Pseudomonas aeruginosa*	5.6	131	129	10.7 (3.25)	10.6 (3.1)	77 (59)	70 (71)	Dornase alfa, tobramycin, ibuprofen, and hypertonic saline
Stick 2022 [24]	RCT	Infants with cystic fibrosis	36	68	62	3.6 (1.2) months	3.6 (1.3) months	38 (56)	40 (65)	NR
Mayer-Hamblett 2018 [21]	RCT	Early *Pseudomonas* infection in cystic fibrosis	11	110	111	7.1 (5.1)	6.8 (5)	55 (50)	62 (55.9)	Ivacaftor/lumacaftor
Shmarina 2017 [23]	Cohort	Pediatric patients with cystic fibrosis	12	59	102	14.3 (0.4)	13 (0.4)	28 (47.5)	59 (57.8)	NR
Klingel 2019 [25]	Cohort	Children with cystic fibrosis with exacerbations who are on tobramycin	12	31	36	13.6 (10.6 to 15.4) median (IQR)	14.9 (12.3 to 16.1)	NR	NR	Tobramycin, colistimethate, amikacin, and aztreonam
Abdul Aziz 2020 [22]	Cohort	Children with cystic fibrosis with exacerbations who are on tobramycin	12	30	33	14.4 (3)	9.96 (3.57)	18 (60%)	21 (63.6)	NR
Saiman 2012 [26]	RCT	Patients with cystic fibrosis uninfected with *Pseudomonas aeruginosa*	5.6	77	69	6–18 years		NR	NR	NR

AZN: azithromycin, RCT: randomized controlled trial, NR: not reported, SD: standard deviation.

## Data Availability

The datasets analyzed during the current study are available from the corresponding author upon reasonable request.

## Supplementary Materials

The following supporting information can be downloaded at: https://www.mdpi.com/article/10.3390/medicina61040653/s1. File S1. Prisma 2020 checklist.
